# A comprehensive review of alkaloids in cancer therapy: focusing on molecular mechanisms and synergistic potential of piperine in colorectal cancer

**DOI:** 10.1007/s13205-025-04535-8

**Published:** 2025-10-27

**Authors:** Chinthana Chidananda, Goutam Thakur, Deepanjan Datta, Ketul Popat

**Affiliations:** 1 Department of Biomedical Engineering, Manipal Institute of Technology, Manipal Academy of Higher Education, Manipal, Udupi, Karnataka 576104 India; 2 Department of Pharmaceutics, Manipal College of Pharmaceutical Sciences, Manipal Academy of Higher Education, Manipal, Udupi, Karnataka 576104 India; 3 Department of Bioengineering, College of Engineering and Computing, George Mason University, Fairfax, VA 22030 USA

**Keywords:** Piperidine alkaloid, Alkaloids, Piperine, Black pepper, Colorectal cancer, Combination therapy, Nutraceuticals

## Abstract

Alkaloids exhibit a wide range of anticancer activities, including the induction of apoptosis, regulation of autophagy, arrest of the cell cycle, inhibition of angiogenesis, and disruption of oncogenic signaling pathways. Among these compounds, piperine, a piperidine alkaloid derived from black pepper, demonstrates multifaceted activity against colorectal cancer (CRC). Preclinical studies indicate that piperine induces apoptosis through mitochondrial and reactive oxygen species (ROS)-mediated mechanisms, arrests the cell cycle at the G_0_/G_1_ and S phases, and suppresses oncogenic signaling pathways, such as Wnt/β-catenin, STAT3/Snail-EMT, and PI3K/Akt/mTOR pathways. Furthermore, it modulates inflammatory and oxidative stress responses by inhibiting NF-κB and activating Nrf2/Keap1 signaling while reducing angiogenesis via Src/EGFR-IL-8 regulation. These multi-targeted actions result in decreased proliferation, migration, invasion, and metastasis of CRC cells. In addition to its intrinsic anticancer properties, piperine serves as a potent natural bio-enhancer. Combination studies revealed synergistic effects with chemotherapeutics (celecoxib, apatinib), radiotherapy, and nutraceuticals (curcumin, resveratrol, and cannabinoids), significantly enhancing therapeutic efficacy and overcoming drug resistance. However, its pharmacokinetic limitations—poor aqueous solubility, rapid metabolism, and low oral bioavailability—pose challenges to its clinical application. Pharmacokinetic analyses reveal high lipophilicity, extensive distribution, and rapid elimination, necessitating innovative strategies to improve systemic stability. Recent advancements include nano-formulations (liposomal, polymeric, and lipid–polymer hybrids) and structural analogs, which increase solubility, stability, bioavailability, and targeted delivery, thereby increasing anticancer efficacy. A comparative evaluation with other alkaloids indicates that while piperine exhibits moderate direct cytotoxicity relative to agents such as camptothecin or berberine, its unique value lies in its broad pathway modulation ability and synergistic potential as an adjuvant. Collectively, mechanistic insights, pharmacologic evaluations, and combinational strategies establish piperine as a promising multifunctional agent for CRC prevention and therapy. Nonetheless, standardized preclinical methodologies, detailed pharmacokinetic profiling, and well-structured early-phase clinical trials are critical to validate its translational potential and facilitate its integration into colorectal cancer management.

## Introduction

**Fig. 1:**
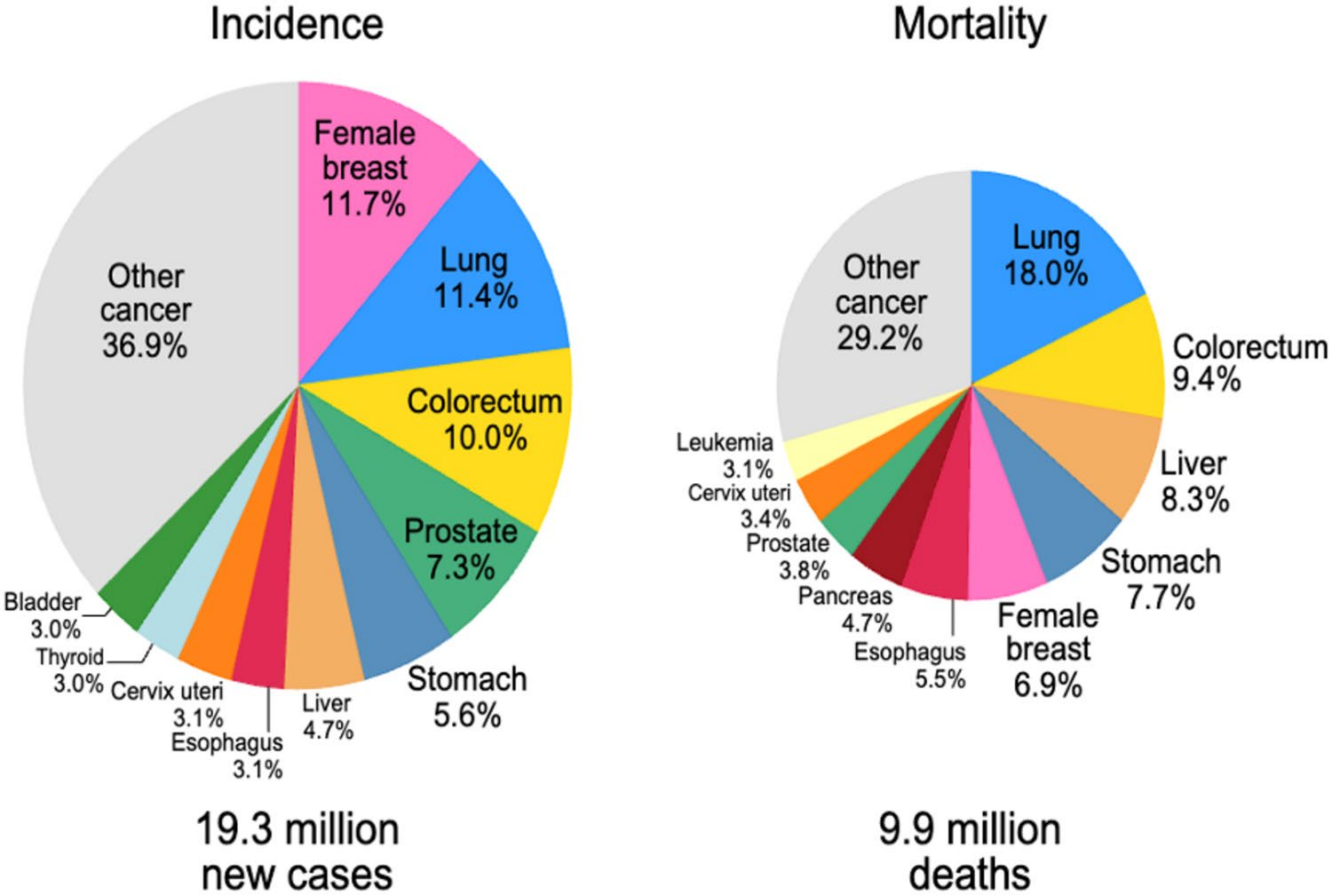
Distribution of Cases and Deaths for the Top 10 Most Common Cancers in 2020 Globally. The image elucidates colorectal cancer (CRC) as a significant global health concern, accounting for 10.0% of new cancer cases and 9.4% of cancer-related mortalities. Despite its classification as the third most prevalent malignancy, the high mortality rate associated with CRC emphasizes the imperative for early screening protocols, advanced therapeutic interventions, and preventive strategies. (Reproduced from [1] Sung et al. 2021, CA: A Cancer Journal for Clinicians, under Creative Commons Attribution License CC-BY)

Colorectal cancer (CRC) is one of the third most diagnosed cancers, accounting for ten percent of all cases worldwide. In terms of mortality, it stands in second place, causing 9.4% of total cancer deaths after lung cancer, as represented in Fig. 1. Among men, it ranks as the third most frequently identified cancer after lung and prostate cancer and second in terms of cancer-related mortality following liver cancer. In women, CRC is the second most commonly diagnosed cancer after breast cancer, and it is the second leading cause of cancer-related mortality after lung cancer and breast cancer (Sung et al. 2021).

In recent decades, CRC treatment has evolved predominantly through the use of a multimodal approach with traditional and emerging targeted therapies. The current treatment comprises a combination of surgical interventions, systematic therapies, and localized treatments tailored to the stage of the disease and patient-specific traits, consequently contributing to improved prognosis and extended lifespan (Benson et al. 2018). Regardless of these treatment advancements, each therapy has its limitations. The overall management of CRC remains a significant clinical challenge, with complications emerging from surgery and toxicity induced by chemotherapy to the limited applicability of targeted therapies (Schirrmacher 2019). Despite advancements in screening and treatment modalities, CRC presents substantial challenges to healthcare systems (Khan And Lengyel 2023).

Therapeutic agents extracted from natural sources have gained importance in the management of various diseases (Chaachouay And Zidane 2024). The incorporation of natural compounds to treat cancer has attracted increasing interest in recent years (Asma et al. 2022). The limitations of existing treatments include alternative methods, specifically the use of natural compounds and ameliorated biomolecules for treatment, aiming to increase bioavailability, stability, and therapeutic efficacy while retaining their inherent pharmacologic benefits (Rodrigues et al. 2021a, b; Rodrigues et al. 2019). These compounds can mitigate adverse effects by efficiently targeting cancer cells while sparing healthy cells (Chartier et al. 2019; Drețcanu et al. 2021). Among many existing natural sources, research on many plants worldwide has revealed the effects of various categories of compounds, including polyphenols, flavonoids, terpenoids, and alkaloids, on their activity via many signaling pathways against CRC (Wang et al. 2022).

A cyclic ring comprising one or more basic nitrogen atoms structurally represents alkaloids (Heinrich et al. 2021). Carl F. W. Meissner, a German scientist, first used the term “alkaloid” in 1819. The Arabic word al-qali, which describes the plant from which soda was initially extracted, is the origin of this term (Croteau et al. 2000). Alkaloids have therapeutic applications in human medicine as anesthetics, cardio-protective agents, anti-inflammatory substances, and anticancer agents (Kurek 2019). Approximately 20% of alkaloids occur as secondary metabolites in plants (Kaur and Arora 2015). As of October 25, 2020, the Dictionary of Natural Products (DNP) recorded 27,683 alkaloids. Between 2014 and 2020, 990 entries were added, representing either newly discovered alkaloids or those that were reexamined from natural sources (Heinrich et al. 2021). This review outlines and further investigates the anticancer potential of alkaloids, particularly focusing on piperine and its role in the treatment of CRC, both alone and in combination with other nutraceuticals and drug molecules.

## Anticancer potential of alkaloids from plant sources

Alkaloids can be classified on the basis of their chemical structure as imidazole, indole, isoquinoline, piperidine, purine, pyrrolidine, pyrrolizidine, quinoline, steroidal, or tropane alkaloids. These nitrogen-containing secondary metabolites exhibit major antineoplastic potential through multiple mechanisms, including the modulation of critical signaling pathways, cell cycle arrest, and angiogenesis inhibition (Olofinsan et al. 2023). Several bioactive alkaloids produced from natural sources have demonstrated significant anticancer activity, acting through mechanisms, such as inducing apoptosis and inhibiting tumor proliferation, highlighting their potential as novel therapeutic agents (Prajapati et al. 2025). Table 1 provides a comprehensive overview of various alkaloid types, their natural sources, representative compounds, chemical structures, cancer types studied, mechanisms of action, and relevant references. Alkaloids can be categorized into different classes, including proto-alkaloids, indole alkaloids, quinoline derivatives, carbazole alkaloids, etc. This table highlights their cytotoxic effects on various cancers, such as breast, colon, lung, and prostate cancer, through mechanisms, such as apoptosis induction, mitochondrial dysfunction, ROS generation, and the inhibition of key signaling pathways.

**Table 1:** Alkaloid types, their plant sources, and anticancer mechanisms

Alkaloid Types	Natural Source	Compounds	Chemical Structure	Cancer studied	Mechanisms studied	References
Proto-Alkaloids	*Colchicum autumnale, Colchicum pusillum*	Colchicine		Breast cancer	Enhanced expression of caspases, p53, and Bax	(Foumani et al. 2022)
				Colon cancer	Downregulation of β-catenin signaling	(Becer et al. 2019)
				Oral cancer	Increased cytosolic Ca^2^⁺ concentration via phospholipase C activation	(Sun et al. 2019)
Cumarine–Alkaloid Conjugate	*Neocryptodiscus papillaris*	Neopapillarine		Renal cancer	Demonstrated selective cytotoxic activity	(Tosun et al. 2020)
Indole Alkaloids	*Rauvolfia reflexa*	Reflexin A		Colon cancer	Caspases 9 induction causes early-stage apoptosis in cells, while caspase 8 activation causes late-stage apoptosis 48 h later	(Fadaeinasab et al. 2020)
	*Alstonia yunnanensis*	Acetoxytabernosine		Hepatocarcinoma	G1 phase DNA replication inhibition and caspase-mediated apoptosis	(Lai et al. 2023)
	*Chaetomium globosum*	Chaetocochin J		Colorectal cancer	Apoptosis and autophagy are caused by downregulating phosphoinositide-3-kinase (PIK3R4), activating phosphorylated AMP-activated protein kinase (AMPK), and encouraging the development of autophagolysosomes	(Hu et al. 2021)
	*Halimeda cylindracea*	Caulerpin		Lung cancer and Colorectal cancer	Limited cell migration, Apoptosis induction and Low toxicity to normal cells	(Dini et al. 2021; Mert-Ozupek et al. 2022)
	*Fascaplysinopsis reticulata*	3,10-dibromofascaplysin		Prostate cancer	Reduced androgen receptor activity and increased enzalutamide sensitivity	(Dyshlovoy et al. 2020)
Quinoline Alkaloid Derivatives	*Annona squamosa*	Coclaurine and 6,7-dimethoxy-1-(α-hydroxy-4-methoxybenzyl)-2-methyl-1, 2, 3,4-tetrahydroisoquinoline (benzylisoquinolines)		Colon, breast and Liver cancer	Cytotoxicity enhanced by hydroxyl groups	(Al-Ghazzawi 2019)
	*Nelumbo nucifera*	Neferine (benzylisoquinolines)		Cervical cancer	ROS generation, Cytochrome c expression and Apoptotic protein expression	(Dasari et al. 2020)
	*Berberis cretica*	Palmatine (isoquinoline)		Breast cancer	Inhibits the estrogen receptors in breast cancer, making MCF-7 cells more sensitive to doxorubicin therapy	(Grabarska et al. 2021)
	*Macleaya cordata*	6-methoxydihydroavicine (Isoquinoline)		Pancreatic and ovarian cancer	Increased production of ROS within cells and disrupted the metabolism of oxaloacetic acid in mitochondria during glycolysis	(Ma et al. 2022; Zhang et al. 2023)
	*Stephania hainanensis*	Crebanine N-oxide		Gastric cancer	Caspases 3 and cytochrome c protein expression cause cell cycle arrest and apoptosis in the G2/M phase	(Wang et al. 2020)
Carbazole Alkaloid	*Murraya koenigii*	Girinimbine		Breast cancer	Inhibited the extracellular signal-regulated kinase/mitogen-activated protein kinase (MEK/ERK) pathway, which caused apoptosis	(Yang and Yu 2021)
		Koenimbine		Ovarian, lung and bladder cancer	Reduce the metabolism of OVCAR3 ovarian cancer cells and PC-3 prostate cancer cells	(Satyavarapu et al. 2020; Xie et al. 2020)
		Mahanimbine			Lower the metabolism of prostate cancer and ovarian cancer cells further Promotes autophagy and reduces p62 protein expression, leading to cell death in bladder cancer cells	
		Mahanine			Induces anoikis in the prostate and ovarian cancer cells via microtubule-associated protein (LC3) induction	
	*Glycosmis pentaphylla (formerly known as Glycosmis arborea)*	Glycosmisines A and B		Liver and Lung Cancer	Concentration-dependent antiproliferation	(Chen et al. 2015)
		3 methoxycarbazole		Breast cancer	Increasing the generation of reactive oxygen species and the expression of the caspase 3 protein causes apoptosis	(Alanazi et al. 2022)
	*Clausena excavate*	Dentatin		Hepatocarcinoma and Colorectal cancer	NF-κb inhibition, Caspase 3/9 expression, G0/G1 cell cycle arrest and Th1-type cytokines elevation	(Andas et al. 2015; Zulpa et al. 2023)
Indoloquinazoline Alkaloid	*Araliopsis soyauxii*	Soyauxinine		Leukemia, Colon and Prostate Cancer	Increased apoptotic caspase expression, elevated ROS levels, and altered mitochondrial membrane potential	(Noulala et al. 2022; Noulala et al. 2020)
Steroidal Alkaloid	*Solanum aculeastrum, Solanum carolinense, Solanum pittosporifolium*	Solamargine		Prostate cancer, gastric cancer, neuroblastoma	Suppression of phosphorylated Akt expression, PI3K/Akt pathway dysfunction, MAPK signaling cascade interference and P-glycoprotein inhibition	(Burger et al. 2018; Fu et al. 2019; Ge et al. 2022)
	*Buxus sempervirens*	Cyclovirobuxine D		Breast cancer	Causes breast cancer cells to undergo autophagy by inhibiting the Akt/mtor signaling pathway, changing LC3 from type I to III, and increasing the expression of ATG5	(Lu et al. 2014)
	*Veratrum californicum*	Cyclopamine		Breast cancer	Hedgehog signaling distortion via Smo protein	(Chai et al. 2013; Turner et al. 2016)
Piperidine Alkaloid	*Piper longum*	Piperlongumine		Breast and Colon Cancer	Modulates GLUT-1 and increases MCT-4 expression, reducing glucose uptake; Enhances doxorubicin-mediated cytotoxicity; Suppresses Bcl-2 protein levels and inhibits the G2/M phase of the cell cycle	(Awasthee et al. 2022; Kumar and Agnihotri 2019)
	*Microcos paniculata*	Microcosamine A		Colon cancer	Antagonism of nicotinic acetylcholine receptors (α4β2, α3β4)	(Still et al. 2013)

## Piperine: piperidine alkaloid

Piperine is a piperidine alkaloid isolated primarily from black pepper with the molecular formula C_17_H_19_NO_3_ (Table 2). Black pepper is the primary source of piperine, accounting for approximately 5–10% by weight, with variations depending on the variety, climate, and drying processes (Ashokkumar et al. 2021). Long pepper (*Piper longum*) has a relatively low piperine concentration, ranging from 1 to 2%, but remains a notable source. Other species within the *Piper* genus, such as cubeb pepper (*Piper cubeba*), are present in much smaller amounts, with cubeb pepper having only approximately 0.1% piperine. Additionally, related peppers, such as Sichuan pepper (*Zanthoxylum simulans*) and paradise grains (*Aframomum melegueta*), contain minimal piperine compared with black pepper (Salehi et al. 2019). This alkaloid functions as an inhibitor of NF-κB (nuclear factor kappa-light-chain-enhancer of activated B cells), exists as a metabolite in plants and human blood serum, and serves as a component of food. It belongs to several chemical categories, including benzodioxoles, *N*-acylpiperidines, piperidine alkaloids, and tertiary carboxamides (National Center for Biotechnology Information, 2025).

**Table 2:** Physicochemical properties of piperine

Physico-chemical properties	Description/value	References
Molecular formula	C₁₇H₁₉NO₃	
IUPAC name	(2E,4E)-5-(1,3-benzodioxol-5-yl)-1-PIPeridin-1yl-penta-2,4-dien-1-one	
Molecular structure		
Molecular weight	285.343 g/mol	(Arora et al. 2023)
Solubility	0.04 mg/mL at 18 °C (poor aqueous solubility in water)	
Log P	2.78	
pKa	1.42 at 55^0^C	(Arnall 1920)
Binding affinity (ΔG)	Human Serum Albumin (HSA): − 7.8 kcal/mol α-1-Acid Glycoprotein (AGP): − 6.71 kcal/mol Brain Tissue Binding: 98.4%–98.5% Plasma protein (96.2–97.8%) Brain-to-Plasma AUC Ratio: 0.95–1.10	(Ren et al. 2018; Yeggoni et al. 2015)
Density	1.193 g/cm^3^	(Arora et al. 2023)
Appearance	Yellow crystalline compound	
Stability	Stable to thermal, neutral and photolytic stress condition	
Half life	Less than 1.5 h	

Piperine has crucial anticancer effects, especially in CRC, by inhibiting cell proliferation and inducing apoptosis in various cancer cell lines without impairing healthy cells. It regulates critical signaling pathways, notably the Wnt/β-catenin pathway, which is often dysregulated in CRC and reduces cell migration and invasion (explained in detail in the coming section and Fig. 6). In addition, piperine has been shown to enhance conventional cancer treatments, such as celecoxib, and nutraceuticals, such as curcumin, suggesting its efficacy as a synergistic agent in cancer therapy. Overall, the multifaceted mechanisms of piperine and its ability to improve existing treatment strategies make it a promising candidate for cancer treatment (Benayad et al. 2023; Bolat et al. 2020; de Almeida et al. 2020; Srivastava et al. 2021).

## Physicochemical properties, pharmacokinetic profiles and molecular docking insights of piperine

The physicochemical properties of piperine, along with its chemical structure, are shown in Table 2. The molar mass of piperine is 285.35 g/mol, and its chemical composition is C_17_H_19_NO_3_. While it has remarkable medicinal properties, it is insoluble in aqueous buffers at 18 °C (approximately 40 mg/l). It is soluble in carbon-based compounds, i.e., organic solvents, such as ethanol, methanol, Transcutol, dimethyl sulfoxide (DMSO), and dimethyl formamide, at a concentration of approximately 10 mg/ml. Owing to its difficulty in water solubility, it has low oral bioavailability and is limited to targeting organs and tissues (Ren et al. 2019; Stasiłowicz et al. 2021).

### Absorption

Piperine is highly lipophilic and follows non-saturable passive absorption kinetics. Studies have revealed limited absorption when piperine is administered intraperitoneally in mice, with a 1–2.5% concentration in the liver and 15% in the spleen, kidney, and serum (Bhat And Chandrasekhara 1986; Quijia et al. 2021). Another study by Suresh and Srinivasan (2010) administered piperine (170 mg kg^−1^) to rats orally, which resulted in a maximum level of 8% of the total amount reaching the intestine after 6 h. In rats, piperine is primarily absorbed in the intestinal tract, with absorption efficiency on the mucosal side of the rat intestine ranging from 40 to 60%. Absorption was noted at higher rates in duodenal segments than in jejunal and ileal segments (Suresh And Srinivasan 2007). Another study in which alkaloids from *Piper longum* L. were orally administered to rats revealed that piperine exhibited rapid absorption, with a mean maximum plasma time (*T*_max_) of 2.45 h, suggesting its effective uptake from the intestinal tract (Liu et al. 2011).

### Distribution

After absorption, piperine is widely distributed throughout the body. Upon oral administration, the concentration of piperine in serum, blood, kidneys, liver, and intestine peaked at 10.8% at the 6-h mark. This concentration decreased to 0.5% after 24 h and further decreased to 0.3% after 48 h. By 96 h, piperine was no longer detectable in any of these tissues (Suresh And Srinivasan 2010). Piperine also restricts hepatic metabolism and effectively enters the brain because of its high binding capacity to brain tissues and plasma proteins (Ren et al. 2018; Yeggoni et al. 2015). In terms of tissue distribution, the liver presented the highest concentration two hours after injection, at approximately 456 ± 291 ng/g (Liu et al. 2013).

### Metabolism

Piperine undergoes extensive metabolism in the body through various reactions, including dehydrogenation, hydrogenation, methylation, glucuronic conjugation, ring cleavage, demethylation, hydroxylation, methoxylation, sulfate conjugation, oxidation, and glucuronidation. These processes result in several metabolites being detected in urine, including piperic acid, piperonylic acid, piperonyl alcohol, piperonal, and vanillic acid. Research has identified 148 metabolites of piperine in mice, indicating the intricate metabolic reactions it undergoes (Shang et al. 2017).

### Excretion

Piperine and its metabolites are essentially excreted through feces and urine. Within the first four days of ingestion, approximately 3.64% of the administered piperine is excreted through feces. After five days, it is no longer detectable in either feces or urine. In particular, approximately 96% of orally administered piperine is absorbed in rats, reflecting its high absorption rate (Suresh And Srinivasan 2010). Piperine has a half-life (*t*_1/2_) of 4.10 h in a rodent model with an administration dose of 54.4 mg/kg (J. Liu et al. 2011), whereas in humans, piperine administration has a *t*_1/2_ of 13.2–15.8 h, implying a relatively slow elimination process (Itharat et al. 2020; Tripathi et al. 2022).

### Bioavailability

Piperine has high lipophilicity and weak basicity, revealing its non-saturable passive absorption kinetics. In 1979, it was also recognized as the first bio-enhancer in the world by Indian scientists at the Regional Research Laboratory in Jammu (Db et al. 2018). Despite its potential, the inability of piperine to dissolve limits both its availability at the site of action and its bioavailability in the body (Quijia et al. 2021). Another study on the oral administration of piperine in rats reported a bioavailability of approximately 37.7% upon the administration of alkaloids from *Piper longum* L. This finding indicates that over one-third of ingested piperine reaches the systemic circulation unaltered, suggesting a moderate value of absorption and metabolism (Liu et al. 2013). A recent study revealed notable increases in the peak plasma concentration (Cmax), area under the curve (AUC), and half-life of traditional drugs when used in conjunction with piperine. These results indicate that piperine might increase drug bioavailability by inhibiting crucial enzymes, such as CYP2 C9, CYP2E1, and CYP3 A4. This holds clinical promise, particularly in boosting the effectiveness of drugs that typically have low bioavailability (Pradeepa et al. 2023).

### Toxicity

As a dietary supplement, piperine generally has relatively low levels of acute and chronic toxicity. When piperine is administered intravenously, it is more toxic than when it is administered intragastrically, subcutaneously, or intramuscularly. The insoluble nature or chemical instability of piperine in the stomach is believed to be the cause of the decreased toxicity observed via the intragastric route. Thus, a greater dosage of piperine results in histopathological lesions in the gastrointestinal tract, mild-to-moderate enteritis in the small intestine, and hemorrhagic ulcers in the stomach, suggesting that piperine has direct and local effects on the gastrointestinal lumen. The LD_50_ values of piperine are listed in Table 3 and are based on the various administration routes used (Piyachaturawat et al. 1983; Zhang et al. 2021). Piperine has not shown genotoxicity in either in vitro or in vivo models (Thiel et al. 2014). It also inhibits spermatogenesis and reduces reproductive capacity in rats at a dosage of 10 mg/kg body weight, highlighting its potential reproductive effect on the administration of higher doses (Chen et al. 2018; Daware et al 2000).

**Table 3:** LD_50_ values based on the route of administration

Mode of administration	LD_50_ in mg/kg body weight	References
Intravenous administration in adult mice	15.1	(Piyachaturawat et al. 1983; Zhang et al. 2021)
Oral administration in mice	330	
Oral administration in rats	514	

The European Food Safety Authority (EFSA) developed a no-observed adverse-effect level (NOAEL) of 5 mg/kg body weight/day from a 90-day rat study, which was used in the Norwegian Scientific Committee for Food Safety’s (VKM) evaluation, which was based on toxicity studies and previous risk assessments. Values of 145 for children, 204 for adolescents, and 234 for adults were obtained via the margin of exposure (MOE) technique; these values were significantly greater than the tolerable cutoff of 100. VKM concluded that there is little chance of pharmacologic or phytochemical interactions at typical dietary levels and that a daily dosage of 1.5 mg of piperine from supplements is unlikely to have a negative effect on the health of children, adolescents, or adults (Rohloff et al. 2018). In human studies, supplemental doses ranging from 4 to 40 mg/day are generally considered safe although reports of mild gastrointestinal discomfort and skin rashes are rare, highlighting the need for ongoing monitoring and further safety evaluations for clinical applications (Ziegenhagen et al. 2021).

### Molecular docking insights into piperine

Molecular docking can be used to virtually screen vast numbers of natural compounds to predict their binding interactions and affinities with a target protein, thereby accelerating the discovery of new therapeutic agents and providing insights into their mechanisms of action (Mishra et al. 2023). These computational studies revealed the significant binding affinity of piperine for various target proteins. Studies have shown that BAX, Cox-2, Caspase 3, and Caspase 9 docked with piperine exhibit binding scores of 3824, 5042, 4174, and 4988 kcal/mol, respectively, suggesting the potential of piperine in colon cancer research (Kirubhanand et al. 2020). Additionally, docking with the EGFR tyrosine kinase exhibited an inhibitory ability with a binding energy of − 7.6 kJ mol^−1^, forming two hydrogen bonds with the PRO699 and ARG831 residues. The docking energy was 7.06, with an inhibitory constant of 2.69e-006 and an intermolecular energy of − 8.22 (Paarakh et al. 2015). These structural characteristics contribute to the ability of piperine to modulate various cellular pathways and increase the bioavailability of co-administered drugs or phytochemicals. These findings align with other molecular modeling studies of natural compounds that target colon cancer via curcumin derivatives as Abl-kinase inhibitors, suggesting that phytochemicals such as piperine warrant further investigation as potential anticancer agents through computational and in vitro validation approaches (Rodrigues et al. 2021a, b).

The effect of the mTOR protein, which is essential for the development of cancer, was further investigated in a computational study. The molecular docking results revealed that piperine had a binding affinity of − 8.3 kcal/mol for the mTOR protein, which is very similar to the binding affinity of rapamycin, a well-known mTOR inhibitor, which is − 8.8 kcal/mol. Piperine binds to the mTOR active site in an ATP-competitive way and remains stable and firm, according to molecular dynamics simulations run over 100 ns. According to the MTT assay results, piperine dramatically reduced the viability of cancer cells, with IC_50_ values of 19.73 ± 0.25 µM at 72 h, 46.3 ± 0.26 µM at 48 h, and 84.5 ± 0.5 µM at 24 h. Although more research is needed, these results imply that piperine is a promising mTOR inhibitor with potential uses in the treatment of cancer (Jan et al. 2025).

### Therapeutic potential of piperine

**Fig. 2:**
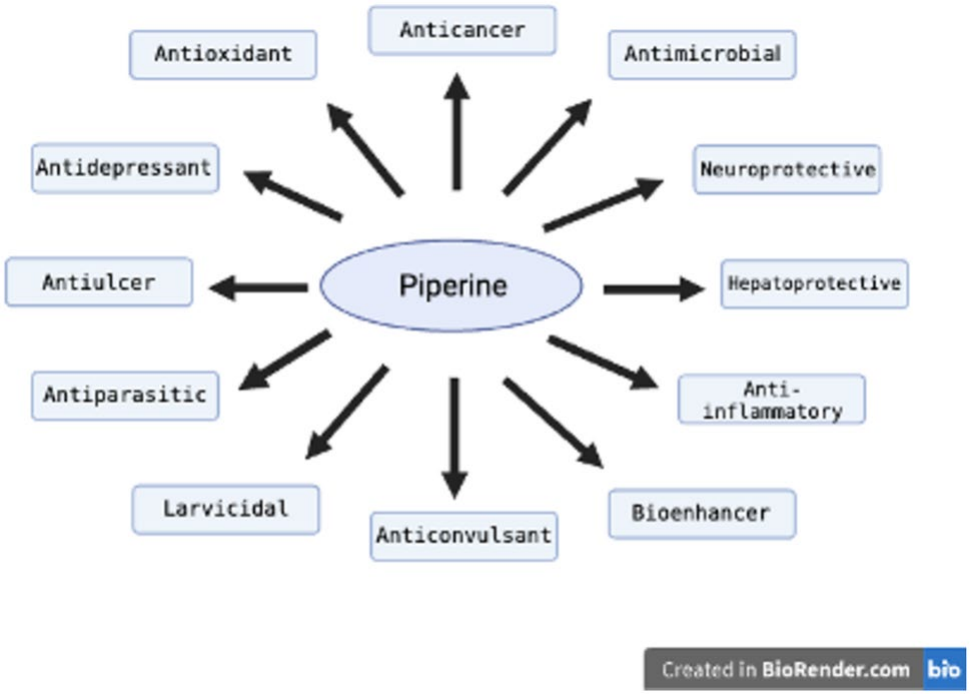
Schematic illustration of Piperine with therapeutic interventions. This image was created under a Creative Commons license using BioRender

In Indian and Chinese medicines, piperine is a significant bioactive substance that has a variety of therapeutic uses, including antibacterial, anticonvulsant, anticancer, and neuroprotective properties (Fig. 2) (Tiwari et al. 2020). As an antioxidant, it effectively reduces reactive substances while maintaining key enzyme activities, such as glutathione and superoxide dismutase (Vijayakumar et al. 2004). Piperine, as an anticonvulsant, delays tonic‒clonic seizures (Mishra et al. 2015), and it is also effective against Gram-positive and Gram-negative bacteria because of its antimicrobial properties (Zarai et al. 2013). As a neuroprotective agent, it reduces cytokine IL-1b expression and improves motor coordination (Chonpathompikunlert et al. 2010), and as a hepato-protective agent, it decreases lipid peroxidation in cells (Gurumurthy et al. 2012). It is also known for its significant anticancer properties, demonstrating its potential to reverse drug resistance in cervical cells (Han et al. 2017). Piperine also has potential in the management of pain, rheumatic arthritis, fever, and influenza (Parthasarathy et al. 2008) but has anti-parasitic properties (Kumar et al. 2012) and larvicidal effects against disease vectors (Samuel et al. 2016). In addition to these traits, it enhances the bioavailability of several drugs, including rifampicin, ibuprofen, and omeprazole (Khatri And Awasti 2016; Khatri et al. 2015), making it valuable in combination therapies.

## Anticancer mechanisms of piperine

**Fig. 3:**
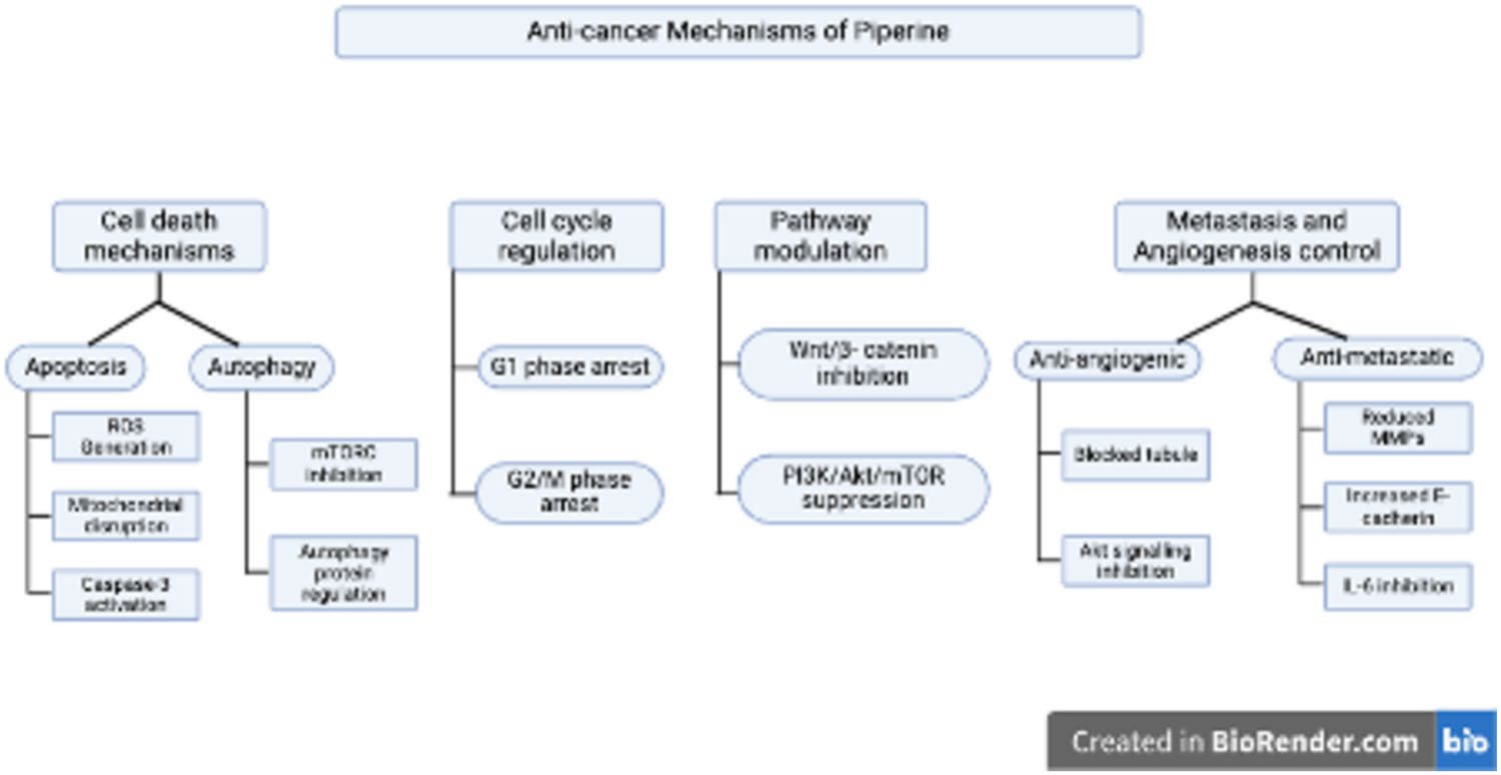
Schematic illustration of anticancer mechanisms of piperine. The figure elucidates piperine’s anticancer mechanisms, encompassing apoptosis (ROS generation, mitochondrial disruption, and caspase-3 activation) and autophagy (mTORC inhibition, protein regulation). It induces G1 and G2/M cell cycle arrest, inhibits Wnt/β-catenin and PI3K/Akt/mTOR pathways, and regulates metastasis and angiogenesis by impeding tubule formation, reducing MMPs, increasing E-cadherin, and inhibiting IL-6. This image was created under a Creative Commons license using BioRender

Piperine exhibits effective anti-inflammatory and apoptotic properties through multiple mechanisms as explained in Fig. 3. It suppresses pro-inflammatory cytokines, such as TNF-α, IL-6, and IL-1β; inhibits inflammatory factors; triggers both intrinsic and extrinsic apoptotic pathways; and exhibits traits, such as nuclear condensation and DNA breakage (Tawani et al. 2016; Wang-Sheng et al. 2017). It increases ROS production by altering the mitochondrial membrane potential and activating caspase-3 (Jafri et al. 2019). Studies have also shown that piperine can trigger autophagy by inhibiting mTORC kinase and controlling proteins linked to autophagy (Kaur et al. 2018; Ouyang et al. 2013). Moreover, altering cyclin and cyclin-dependent kinases induces cell cycle arrest at the G_1_ and G_2_/M phases (Fofaria et al. 2014; Greenshields et al. 2015).

The anticancer action of piperine involves several signaling pathways and anti-metastatic properties. It inhibits the PI3K/Akt/mTOR pathway in oral cancer cells (Han et al. 2023) and suppresses the Wnt/β-catenin pathway in CRC cells (de Almeida et al. 2020). By preventing tubule formation and Akt activation in breast cancer cells, this alkaloid has strong antiangiogenic effects (Doucette et al. 2013). The decrease in tumor nodules in lung metastasis models and the suppression of IL-6 expression in gastric cancer cells are proof of its anti-metastatic action (Greenshields et al. 2015; Lin et al. 2007). By increasing E-cadherin levels and decreasing MMP-9 and MMP-13 expression, piperine also decreases cancer cell invasion while preserving the integrity of the extracellular matrix (Zare et al. 2021). These diverse mechanisms collectively contribute to the potential of piperine as a therapeutic agent in cancer treatment.

## Role of piperine in colorectal cancer (CRC)

**Fig. 4:**
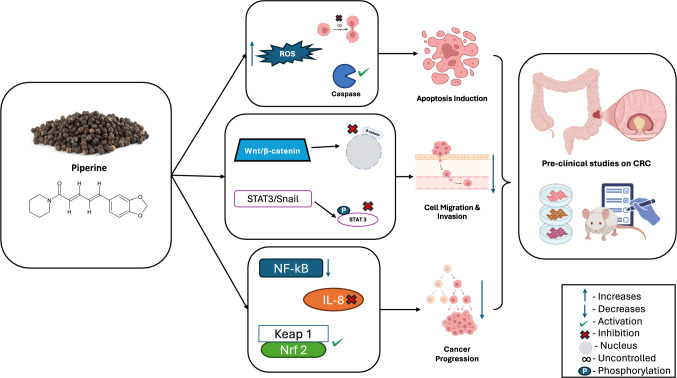
Schematic illustration of multitargeted therapeutic approach of piperine in CRC management. Piperine modulates oxidative stress (↑ROS), suppresses pro-inflammatory (NF-κB, IL-8) and proliferative signals (Wnt/β-catenin, STAT3/Snail), and activates Nrf2-mediated antioxidant response by disrupting Keap1 binding. These actions collectively lead to increased caspase activation and apoptosis, while inhibiting cell migration, invasion, and tumor progression

Piperine can potentially treat CRC through its anti-inflammatory, antioxidant, anti-apoptotic, and many other mechanisms of action by which it can act as an anticancer agent. Studies have shown that it can influence several crucial cellular functions, such as apoptosis activation, cell cycle arrest, and cancer stem cell manipulation, to inhibit tumor development and survival. Focusing on these mechanisms, piperine has the potential to overcome the limitations of conventional treatments and offers a feasible strategy for enhancing the management of CRC. Table 4 highlights the preclinical studies performed on the use of piperine against CRC in various cell lines and animal models. A comprehensive method of cancer treatment involves the molecular mechanisms of piperine in colorectal cancer. Piperine shows great promise as a multifunctional anticancer drug that targets several cellular processes, such as apoptosis, autophagy, oncogenic signaling, and inflammatory responses. Figure 4 highlights the multi-targeted therapeutic approach of piperine studied thus far in preclinical CRC models.

**Table 4:** Preclinical studies on the effects of piperine against CRC

Type of preclinical study	Animal/cell line model used	Signaling pathways involved	References
In vitro	SW480 and HCT-116 cell lines	The study investigated two main pathways: EMT pathway regulation (which showed decreased Vimentin, increased E-cadherin, and suppressed EMT regulator Snail) and STAT3 signaling (which demonstrated decreased STAT3 phosphorylation and inhibited STAT3/Snail-mediated EMT)	(Song et al. 2020)
In vitro	CRC cell lines (HCT116, SW480, DLD-1) and non-tumoral cell line (IEC-6)	Inhibition of β-catenin nuclear localization, Wnt/β-catenin pathway and cell cycle arrest	(de Almeida et al. 2020)
In vivo	4–6-week-old male Wistar rats (DMH-induced)	The study investigates the regulation of the Nrf-2/Keap-1 signaling pathway, the inhibition of NF-κB, and the modulation of inflammatory mediators and oxidative stress markers	(Rehman et al. 2020)
In vitro	HCT116 and HT29 cell lines	Piperine inhibited cell proliferation, induced apoptosis, arrested cell cycle in the S-phase, and reduced EMT markers by downregulating ARL3 expression, thereby attenuating endoplasmic reticulum stress and disrupting EMT induced by TGF-β	(Wu et al. 2025)
In vitro and in vivo	Human CRC cell lines (HCT116, SW480, SW620, DLD-1) and mouse model (CT26 cells)	Inhibition of AKT/mTOR pathway and ROS production	(Xia et al. 2024)
In vitro	HCT-116, HT-29, SW-620, and SW-480 cell lines	Inhibition of IL-8 expression through Src/EGFR-mediated ROS signaling, affecting angiogenesis	(Li et al. 2022)
In vitro	HRT-18 human rectal adenocarcinoma cells	Induction of apoptosis via reactive oxygen species	(Yaffe et al. 2013)
In vitro	HT-29, Caco-2, SW480, HCT-116 colon cancer cell lines	G1 phase cell cycle arrest, apoptosis via ER stress	(Yaffe et al. 2015)
In vitro	SW480 cell line	The study investigates the induction of autophagic cell death and the upregulation of the p53 signaling pathway	(Shao and Wu 2021)

### Molecular mechanisms of piperine in the treatment of CRC

#### Induction of apoptosis

Inducing apoptosis in cancer cells is a crucial action in which piperine works as an anticancer agent. Yaffe et al. (2013) reported that reactive oxygen species (ROS) production is the primary cause of the complex initiation of cell death mechanisms in HRT-18 cells, which are derived from human colorectal adenocarcinoma of the ileocecal region and are used for studying the anticancer effects of piperine. Significant cellular changes, such as cell growth suppression, cell cycle arrest at the *G*_0_/*G*_1_ phase, and the production of damaging hydroxyl radicals, are the main attributes of this mechanism. In colorectal cancer (CRC), the mitochondrial apoptotic pathway eliminates cancer cells by releasing cytochrome c from the mitochondria. This release triggers the activation of caspase-9, followed by caspase-3, ultimately resulting in cell death. This sequence of events effectively overcomes the defense systems of cells, including anti-apoptotic proteins such as survivin, which is upregulated by the phosphoinositide 3-kinase (PI3K)/AKT signaling pathway to increase cell survival and resistance to treatment (Abraha And Ketema 2016; Leiphrakpam And Are 2024). The PI3K/AKT/mTOR pathway is a crucial signaling network that, when disrupted, promotes increased cellular growth, survival, and metabolism, thereby giving cancer cells a proliferative edge (Li et al. 2024). A study revealed that piperine triggered the mitochondrial apoptotic pathway, which is characterized by the release of cytochrome c, the activation of caspase-9 and caspase-3, and the suppression of survival pathways. This multistep process efficiently removes the cancer cell’s defense mechanisms by decreasing the expression of survivin and disrupting the PI3K/Akt signaling pathway (Yaffe et al. 2015).

**Fig. 5:**
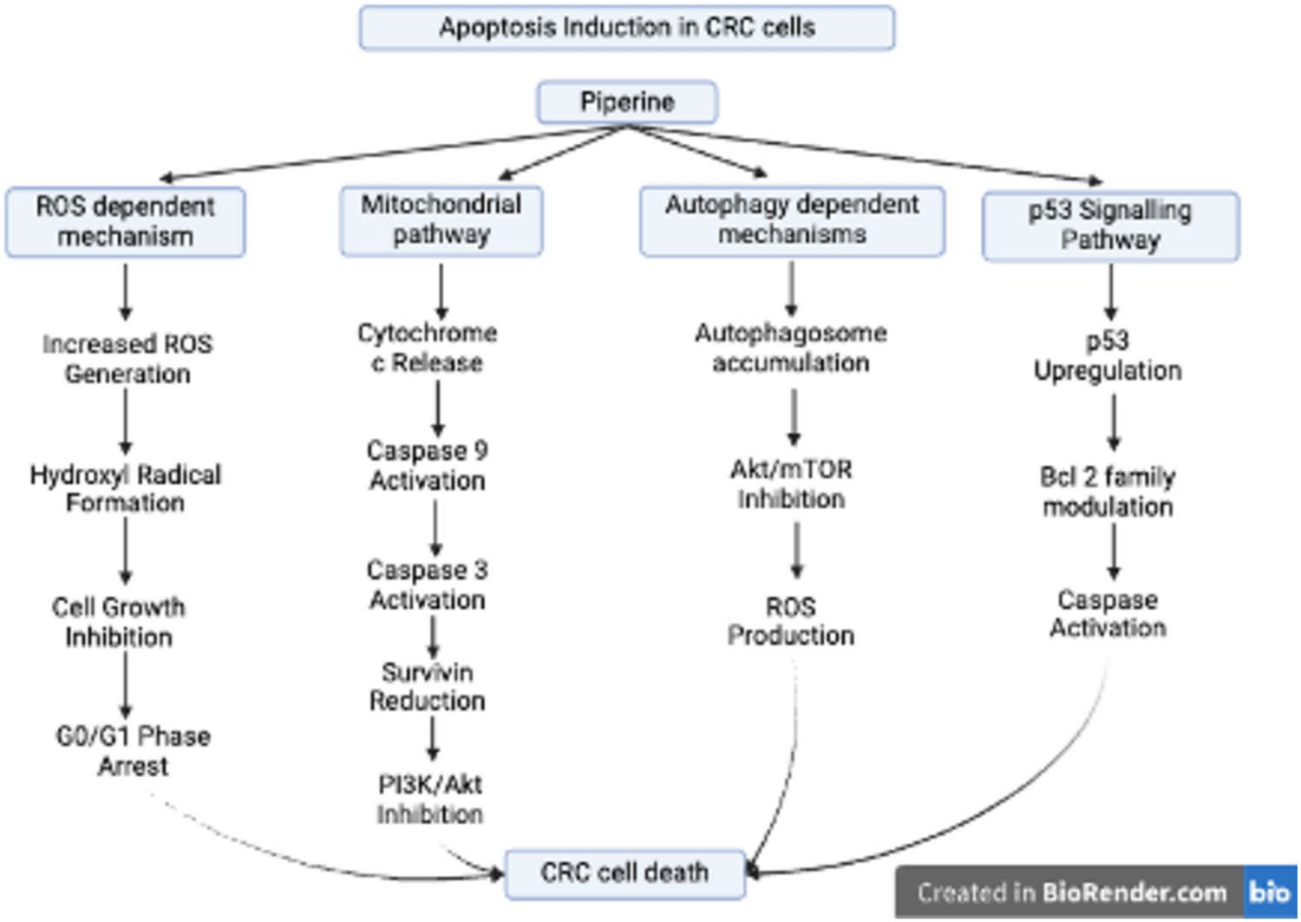
Apoptosis Induction by Piperine in CRC Cells. The figure demonstrates piperine-induced apoptosis in colorectal cancer (CRC) cells through four primary pathways: a reactive oxygen species (ROS)-dependent mechanism (characterized by increased ROS, hydroxyl radical formation, and G0/G1 arrest), a mitochondrial pathway (involving cytochrome c release, caspase activation, and PI3K/Akt inhibition), autophagy-dependent mechanisms (including autophagosome accumulation, Akt/mTOR inhibition, and ROS production), and a p53 signaling pathway (encompassing p53 upregulation, Bcl-2 modulation, and caspase activation), all of which culminate in CRC cell death. This image was created under a Creative Commons license using BioRender

Recent research has revealed that piperine can lead to autophagy-dependent cell death in CRC cells in addition to conventional apoptosis. These alternate methods include auto-phagosome accumulation, AKT/mTOR signaling pathway suppression, and increased reactive oxygen species generation. These mechanisms have minimal effects on healthy colonic epithelial cells and exhibit exceptional selectivity toward cancer cells (Xia et al. 2024). These findings are further supported by (Shao and Wu 2021), who reported that piperine upregulates the p53 signaling pathway and alters the equilibrium of Bcl-2 family proteins by activating caspases. Figure 5 summarizes the mechanism of apoptosis identified in these studies in detail.

#### Oncogenic pathway inhibition

CRC is driven by various oncologic signaling pathways, including the Wnt/β-catenin, RAS/RAF/MEK/ERK, transforming growth factor-beta (TGF-β) circuits and signal transducer and activator of transcription 3 (STAT3/Snail pathway) (Li et al. 2024). The Wnt/β-catenin signaling pathway is the central mechanism involved in the development and progression of CRC because it is involved in cellular growth, stem cell renewal, angiogenesis, epithelial‒mesenchymal transition (EMT), metastasis, and chemo-resistance (He And Gan 2023). Mutations in pathway components, specifically tumor suppressor adenomatous polyposis coli (APC), lead to the nuclear accumulation of β-catenin and the activation of target genes that further support the proliferation and survival of CRC cells. Mutation or loss of APC function leads to uncontrolled cell growth and is strongly associated with the development of colorectal cancer (Noe et al. 2021). The epithelial‒mesenchymal transition (EMT) is a process that converts polarized epithelial cells into mesenchymal cells, which are known for their increased mobility and invasive capabilities. EMT plays a crucial role in the metastasis and progression of colorectal cancer (CRC), particularly in terms of invasion, internal infiltration, and colonization. In addition to promoting metastasis, EMT aids cancer cells in adapting to various microenvironments, acquiring stem cell-like traits, undergoing metabolic changes, and evading treatments (Nie et al. 2025). The STAT3/Snail pathway is a significant regulator of EMT in CRC, with STAT3 enhancing the expression and stabilization of Snail, a key transcription factor that suppresses epithelial markers and promotes mesenchymal characteristics (Hashemi et al. 2023). This STAT3/Snail pathway contributes to tumor invasion and metastasis and is also associated with the development of cancer stem cell properties, further increasing CRC aggressiveness and resistance to therapy (Gargalionis et al. 2021).

**Fig. 6:**
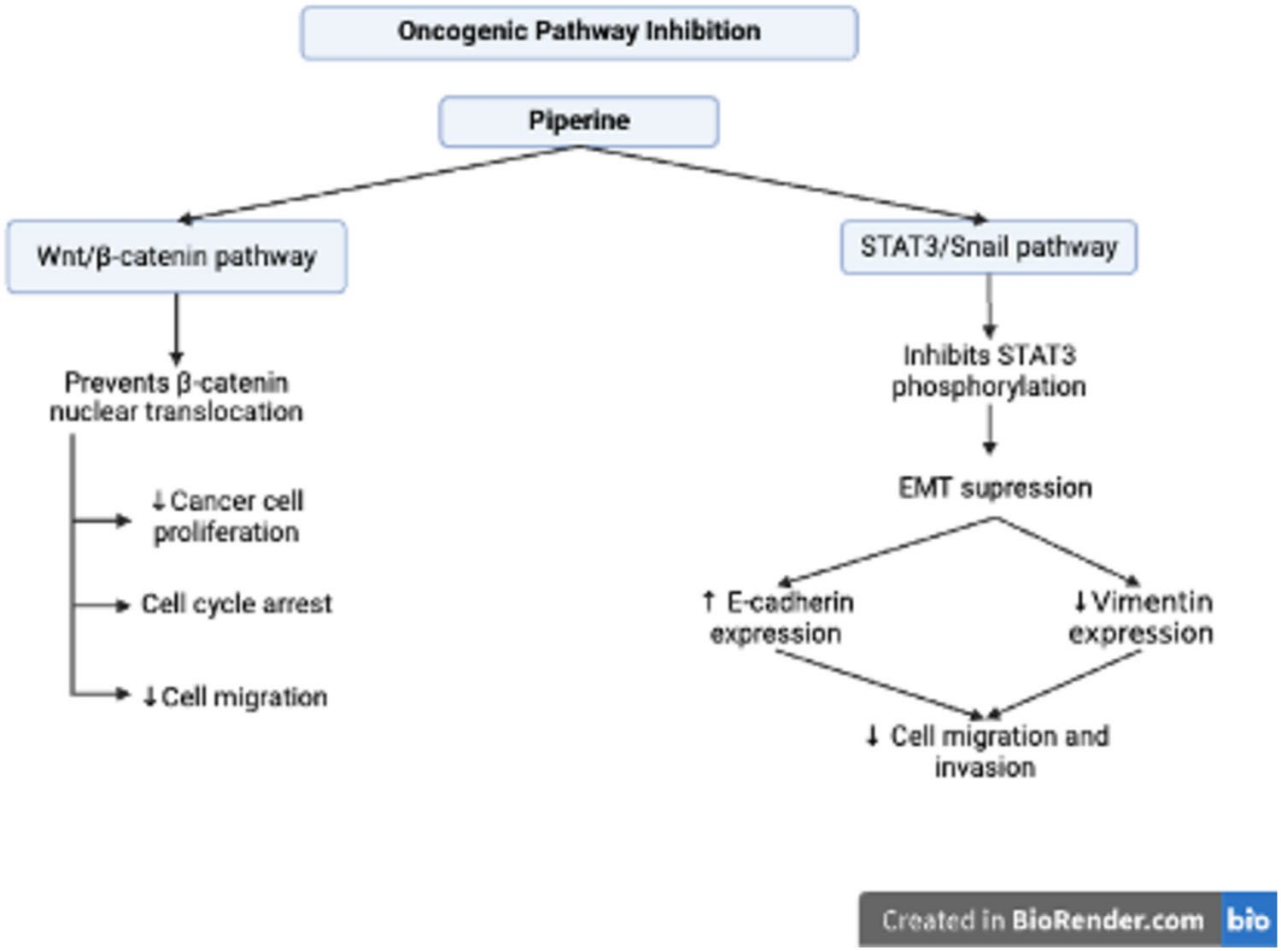
Oncogenic Pathway Inhibition by Piperine in CRC Cells. Piperine inhibits oncogenic pathways through the targeting of Wnt/β-catenin and STAT3/Snail signaling. It impedes β-catenin nuclear translocation, thereby reducing cancer cell proliferation, inducing cell cycle arrest, and decreasing migration. Furthermore, piperine inhibits STAT3 phosphorylation, thus suppressing epithelial–mesenchymal transition (EMT) by increasing E-cadherin expression and reducing Vimentin expression, ultimately limiting colorectal cancer (CRC) cell migration and invasion. This image was created under a Creative Commons license using BioRender

The anticancer potential of piperine includes the inhibition of significant oncogenic signaling pathways, which are illustrated in detail in Fig. 6. According to de Almeida et al. (2020), piperine can block the Wnt/β-catenin signaling pathway, which is a major growth factor in cancer cells. It also selectively inhibits CRC proliferation by inducing cell cycle arrest and hindering cellular migration by blocking β-catenin nuclear translocation. Another study focused on the impact of piperine on epithelial‒mesenchymal transition (EMT), a key step in cancer metastasis. It inhibits the invasion and migration of cells via the STAT3/Snail pathway. This is accomplished by impeding STAT3 phosphorylation, decreasing the production of mesenchymal markers such as vimentin, and increasing the expression of E-cadherin (Song et al. 2020).

#### Inflammatory and stress response modulation

Inflammatory and stress response pathways play crucial roles in the development and progression of CRC through signaling modules such as the Nrf2/Keap1 pathway; the NF-κB and lithocholic acid (LCA)-induced Src/epidermal growth factor receptor (EGFR) signaling pathways; and the JAK/STAT and COX2/PGE_2_ pathways. These factors affect tumor microenvironment, metabolism, and immune system (Mariani et al. 2014; Wei et al. 2022; Li And Huang 2024). The Nrf2/Keap1 pathway in colorectal cancer has dual functions: it typically shields colon cells from oxidative stress and inflammation by enhancing antioxidant and detoxification enzymes, thereby lowering cancer risk. However, when it is overexpressed, it can aid in tumor survival, metastasis, and resistance to chemotherapy, making its balanced regulation essential for both cancer prevention and treatment strategies (Lee et al. 2021). The NF-κB pathway is crucial in CRC as it regulates inflammation, cell proliferation, apoptosis, angiogenesis, metastasis, and drug resistance. It is often persistently activated in CRC cells, fostering tumor growth and survival while enabling cancer cells to evade cell death. NF-κB functions by increasing the levels of pro-inflammatory cytokines (e.g., TNF-α, IL-1β, and IL-6) and anti-apoptotic proteins, contributing to a pro-tumorigenic microenvironment. Its activation is associated with chemotherapy resistance, making NF-κB a potential therapeutic target in CRC (Bahrami et al. 2024). Additionally, pathogen-induced activation of NF-κB (such as by *Fusobacterium nucleatum*) can further drive CRC progression through inflammation and cell invasion mechanisms (Galasso et al. 2025).

Lithocholic acid (LCA), a secondary bile acid, induces interleukin-8 (IL-8) expression in CRC cells, particularly through the activation of the Src/epidermal growth factor receptor (EGFR) signaling pathway. LCA-induced activation of Src and EGFR triggers downstream signaling via the ERK1/2 and AKT pathways, which increase IL-8 expression. This upregulation of IL-8 promotes angiogenesis and tumor progression in CRC. Notably, inhibiting EGFR and Src activity, such as with compounds such as piperine, can suppress LCA-stimulated IL-8 expression, reducing CRC cell angiogenesis and invasiveness. Thus, LCA promotes CRC progression through Src/EGFR-driven IL-8 expression, and targeting these pathways may reduce tumor growth and metastasis (Li et al. 2022; Nguyen et al. 2017).

**Fig. 7:**
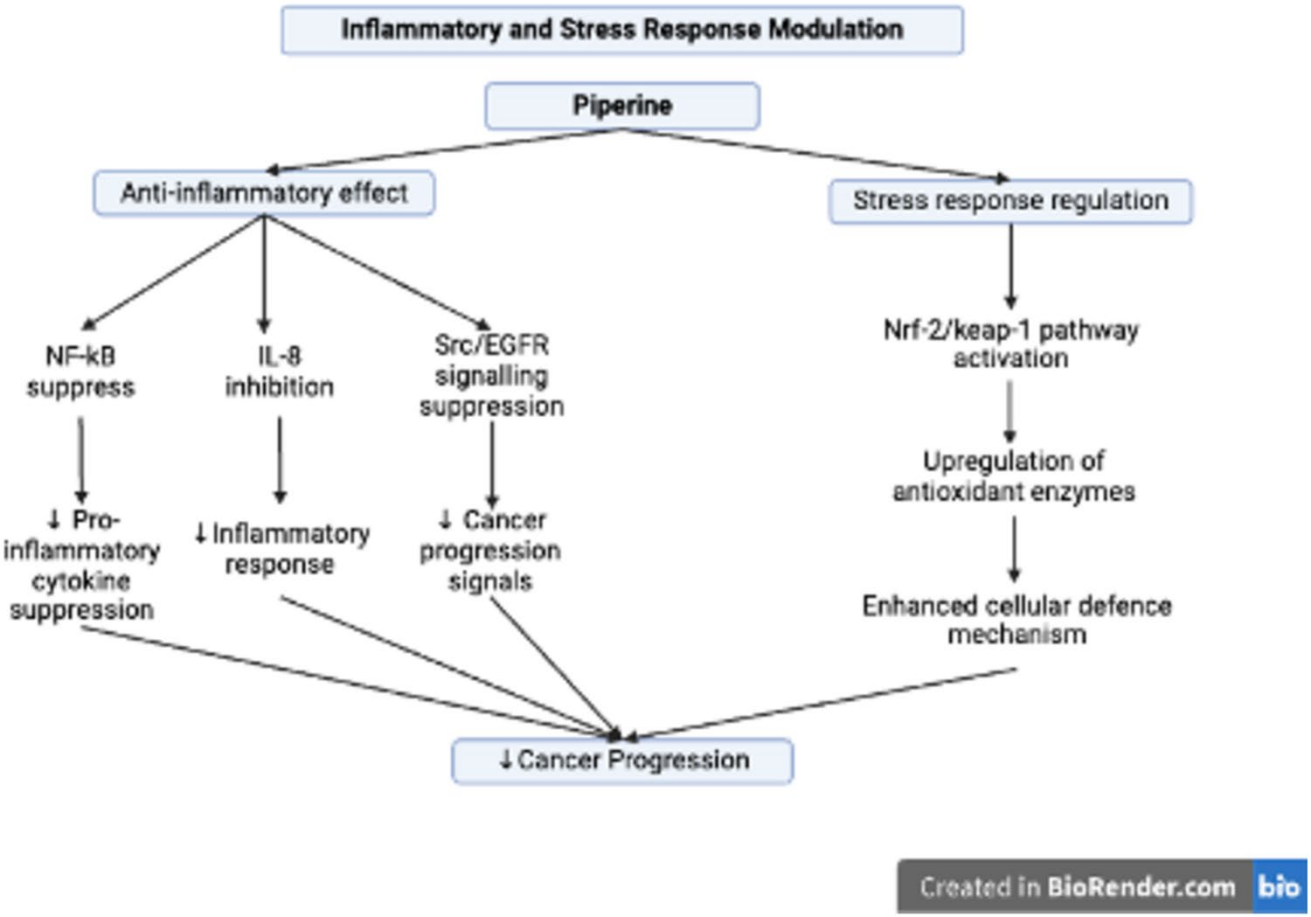
Inflammatory and stress response modulation by piperine in CRC cells. Piperine modulates inflammatory and stress responses to inhibit colorectal cancer (CRC) progression. It exerts anti-inflammatory effects by suppressing nuclear factor kappa B (NF-κB), inhibiting interleukin-8 (IL-8), and downregulating Src/epidermal growth factor receptor (EGFR) signaling, resulting in reduced cytokine production, inflammatory response, and cancer progression signals. Furthermore, it regulates stress response by activating the nuclear factor erythroid 2-related factor 2/Kelch-like ECH-associated protein 1 (Nrf-2/Keap-1) pathway, upregulating antioxidant enzymes and enhancing cellular defense mechanisms, thereby further contributing to CRC inhibition. This image was created under a Creative Commons license using BioRender

**Fig. 8:**
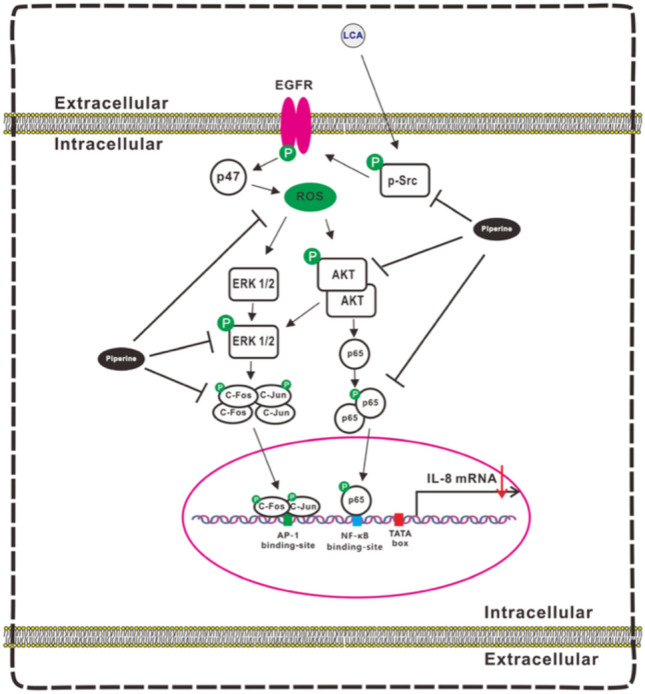
Diagram Illustrating the Inhibitory Mechanism of Piperine on LCA-Triggered IL-8 Production in CRC Cells and Its Impact on CRC-Derived Angiogenesis in the Surrounding Environment. In human colorectal HCT-116 cells, LCA stimulated IL-8 expression by enhancing the transcriptional activity of AP-1 and NF-κB through Src/EGFR-mediated ROS signaling pathways. Piperine inhibited LCA-induced IL-8 expression by suppressing the transcriptional activity of AP-1 and NF-κB and attenuating the Src/EGFR-mediated ROS-activated ERK1/2 and AKT signaling pathways. (Reproduced from [64] Li et al. 2022, antioxidants, under Creative Commons Attribution License CC-BY)

The influence of piperine on inflammatory pathways and cellular stress responses (Fig. 7) highlights that it enhances cellular defense mechanisms and the activity of antioxidant enzymes through the Nrf-2/Keap-1 pathway. Moreover, it inhibits NF-κB activation, further decreasing the expression of mediators and pro-inflammatory cytokines that aid in cancer development (Rehman et al. 2020). Piperine also inhibits lithocholic acid (LCA)-stimulated interleukin-8 (IL-8) expression by suppressing epidermal growth factor receptor (EGFR) and Scr in HCT-116 cells (Li et al. 2022). Figure 8 summarizes the mechanism underlying the inhibitory effect of piperine on LCA-induced IL-8 expression in HCT-116 cells and its effect on CRC-derived angiogenesis in the tumor microenvironment.

### Potential of piperine in combinatorial therapies

As discussed earlier, piperine is considered a natural bio-enhancer for exploring this property, and many preclinical studies have been conducted over the years with a wide range of compounds, including chemotherapeutic drugs, radiation, and nutraceuticals. Table 5 summarizes the key research examining the synergistic role of piperine in combination therapy in CRC models.

**Table 5:** Preclinical studies on the use of piperine in combination therapies

Compounds studied along with piperine	Type of preclinical study	Animal/cell line model used	Signaling pathways involved	References
Curcumin and cannabinoid compounds	In vitro	HCT116 and HT29 cell lines	Piperine enhances the bioavailability and therapeutic efficacy of curcumin The combination with cannabinoids (CBD and CBG) shows improved anticancer effects compared to individual compounds	(Yüksel et al. 2023)
Celecoxib (CXB)	In vitro and in vivo	HT-29 cell lines and Adult female BALB/c mice in which CT26.WT (5 × 106) cells were inoculated subcutaneously	Modulation of Wnt/β-catenin signaling pathway, induction of apoptosis, and generation of reactive oxygen species (ROS)	(Srivastava et al. 2021)
Radiation	In vitro	HT-29 (human colon cancer cell line)	Piperine sensitizes cancer cells to radiation, inducing apoptosis through the mitochondrial pathway, G2/M arrest, and increasing ERβ expression	(Shaheer et al. 2020)
Apatinib	In vitro	HCT-116 cell line	Effects on MDM-2 gene expression, glutathione peroxidase activity, and nitric oxide levels	**(**Mohammadian et al. 2022)
Curcumin	In vitro	HCT116 colorectal cancer cell line	The study examined cell morphology, cell viability, cellular uptake, apoptotic cell death, cell cycle analysis, and caspase-3 gene expression levels to understand how curcumin and piperine interact with HCT116 cancer cells	(Bolat et al. 2020)
Curcumin	In vitro and in vivo	Caco-2 cell line and DMH-induced CRC mice model	COX-2 and iNOS inhibition	(Slika et al. 2020)
(−)-Epigallocatechin-3-gallate	In vivo	Male CF-1 mice	Inhibition of glucuronidation of EGCG by small intestinal microsomes and alteration of gastrointestinal transit	(Lambert et al. 2004)
Resveratrol	In vitro	B16F10 and CT26 cancer cell lines	The study investigated the enhancement of radiosensitivity through increased production of reactive oxygen species (ROS) and the induction of apoptosis in cancer cells	(Tak et al. 2012)
Pentagamavunone-1 with Diosmin, Galangin	In vitro	WiDr colon cancer cell line	The study investigated the cytotoxic effects and potential synergistic effects of the combination of PGV-1 with diosmin, galangin, and piperine, focusing on their interactions with overexpressed proteins in colon cancer	(Ikawati et al. 2023)

#### Conventional therapies

Over the last decade, administering anticancer medications to cancer patients with stage III and IV CRC, either alone or in combination with other drugs, has significantly improved their chances of living longer (Anand et al. 2023). Research has shown that piperine can reduce the therapeutic dose of chemotherapeutic medications, making them less hazardous because of additive or synergistic effects. Through the control of several signaling pathways, piperine has been acknowledged for its substantial contributions to the treatment and management of a variety of malignancies, thereby addressing the issue of chemo-resistance (Manickasamy et al. 2024). In one study, piperine increased the oral bioavailability of celecoxib both in vitro and in vivo by acting as a bio-enhancer. In HT-29 cells, this combination decreased the expression of stemness markers and increased cytotoxicity, ROS generation, caspase activation, apoptosis, and mitochondrial dysfunction. A mechanism involving the suppression of COX-2 activity mediated this synergistic effect. The reduction in HT-29 cell tumorosphere-forming ability and the expression of stemness markers suggest other mechanisms by which this synergism prevents tumor formation. Additionally, in vivo research has shown that induced β-catenin degradation and Axin stabilization adversely regulate Wnt/β-catenin signaling molecules and simultaneously halt the cell cycle at the G_1_ phase by lowering cyclin D1 (Srivastava et al. 2021).

The effectiveness of piperine in increasing the radio-sensitivity of colorectal cancer cells (HT-29) was examined in another study. Following treatment with piperine, the HT-29 cells were exposed to 1.25 Gy of gamma radiation. To clarify the mechanisms of this combined treatment in contrast to each treatment given separately, researchers have investigated a number of pathways via flow cytometry, immunofluorescence, and immunoblot assays. The combination treatment halted HT-29 cells in the G_2_/M phase 2.8 times more efficiently than did radiation alone, causing cell death via a mitochondria-dependent route. There was a noticeable increase in key cell death mechanisms, such as caspase-3 activation and poly(ADP‒ribose) polymerase-1 cleavage. The activation of estrogen receptor beta (ERβ), which aids in tumor suppression, represents a recent advancement in the treatment and prevention of cancer. Overall, by preventing DNA damage, apoptosis, and cancer cell line proliferation, piperine increases the radio-sensitization of colon cancer cell lines (Shaheer et al. 2020).

In a different study, scientists reported that when apatinib and piperine were administered together, HCT-116 cells presented reduced glutathione peroxidase activity, decreased MDM2 gene expression, decreased cell viability, and increased nitric oxide levels. By examining the MDM-2 gene expression ratio, the possible harmful mechanisms of these substances were investigated. As either the piperine or apatinib concentration increased, the rate of CRC cell growth decreased. Synergistic effects were observed when HCT-116 cells were treated with different doses of piperine and apatinib. Compared with those in untreated control cells and those subjected to individual treatments, MDM-2 gene expression was downregulated, and nitric oxide (NO) levels were increased in cells treated with both apatinib and piperine. Furthermore, compared with that in the individual treatments, the glutathione peroxidase activity was considerably lower in the combined treatment. The proliferation of CRC cells was strongly inhibited by the combination of piperine and apatinib. The inhibition of antioxidant indicators and the control of MDM-2 gene expression caused these harmful effects (Mohammadian et al. 2022).

#### With other natural compounds

Piperine exhibits synergistic potential when combined with other natural compounds for CRC treatment, particularly in strengthening its anticancer potential by increasing cell death, inhibiting proliferation, and modulating key signaling pathways. Using human HT29 and HCT116 colon cancer cell lines, piperine was combined with curcumin and cannabis derivatives (cannabidiol/cannabigerol) to show therapeutic potential in colon carcinoma. This triple combination activated the Hippo/YAP signaling system, which caused apoptosis and decreased cell viability (Yüksel et al. 2023). Curcumin and piperine were combined to create emulsomes to increase their limited bioavailability and produce a synergistic anticancer effect in an in vitro colon cancer model. By analyzing cell shape, viability, uptake, apoptotic cell death, the cell cycle, and caspase-3 gene expression levels, the effects of the combined therapy were evaluated, and insights into the molecular interactions of these active chemicals with HCT116 cancer cells were obtained. Aggregation, a spherical form, and shrinking are morphological alterations that suggest the activation of apoptosis. Further evidence of apoptosis induced by the combined therapy was provided by the observation of cell cycle arrest at the G_2_/M phase and an increase in the number of Annexin V-positive cells. The anticancer activity of curcumin and piperine emulsomes is enhanced by a six-fold increase in caspase-3 gene expression levels (Bolat et al. 2020).

Researchers have shown that the combined use of piperine and curcumin can decrease the activity of tumor necrosis factor α (TNF-α) signaling and mammalian target of rapamycin complex 1 (mTORC1) in the intestinal lining, suggesting that it has great potential to address inflammation and cancer development in patients with colorectal cancer. Whether taken independently or in combination, CUR and PIP can inhibit intestinal lining mTORC1 signaling, which may have consequences for the development of cancer and inflammatory diseases. Compared with CUR, PIP was found to be a less effective inhibitor of mTORC1 in HT-29 and Caco-2 cells, except in differentiated Caco-2 cells. Compared with CUR alone, the combination of CUR and PIP resulted in more pronounced inhibition of mTORC1, indicating an additive effect. Given that PIP independently suppresses mTORC1 and may also increase the effectiveness of CUR by increasing its bioavailability, the interaction between PIP and CUR appears to be more intricate (Kaur et al. 2018). Piperine has the potential to increase the bioavailability of EGCG, a compound found in green tea. Mechanistically, piperine seems to inhibit the glucuronidation of EGCG in the small intestine, which could lead to increased absorption. Additionally, piperine slows the gastrointestinal transit of EGCG, thereby extending its residence time in the intestine and facilitating greater absorption. The increased plasma bioavailability of EGCG and piperine could increase their cancer-preventive effects in vivo (Lambert et al. 2004).

Piperine and resveratrol exhibit anticancer effects on B16F10 melanoma and CT26 colon carcinoma cell lines by facilitating apoptosis and suppressing cell proliferation. An increase in cellular damage, which leads to cell cycle arrest and the activation of apoptosis-related proteins such as caspase-3, increases the effectiveness of cancer treatments such as *γ*-irradiation. Furthermore, both substances have the ability to alter mitochondrial activity and produce reactive oxygen species (ROS), which help destroy cancer cells (Tak et al. 2012). Piperine, when combined with PGV-1, a curcumin analog, enhances anti-colon cancer activity. These findings demonstrate that piperine significantly synergizes with PGV-1 at low concentrations, leading to increased suppression of colon cancer cell viability. Piperine’s targeting of particular proteins that are overexpressed in colon cancer cells—particularly AURKA (Aurora kinase A) and CDK1, which are important regulators of cell cycle progression—is what causes this synergy. According to protein prediction analyses, piperine and PGV-1 share overlapping targets, especially CDK1, which suggests that piperine may block cell cycle regulators and thus promote apoptosis and cell cycle arrest. Additionally, the overexpression of other target proteins, such as AURKA and CDK1, is correlated with poorer patient survival, indicating that disrupting these pathways with combination therapies can be effective (Ikawati et al. 2023). In future, it will be necessary to conduct more preclinical and clinical studies using piperine in combination with other available therapeutic regimens to evaluate its pharmacodynamic and pharmacokinetic interactions with standard chemotherapeutic agents and other natural compounds, such as nutraceuticals.

## Comparison of the roles of piperine with those of other alkaloids

Comparing the activity of piperine with that of other well-known alkaloids is necessary because alkaloids are among the most diverse and extensively studied classes of phytochemicals. By producing reactive oxygen species (ROS), inhibiting the Wnt/β-catenin and STAT3 pathways, reversing the epithelial–mesenchymal transition (EMT), and other mechanisms, piperine has anticancer effects on colorectal cancer (CRC) models (Song et al. 2020; de Almeida et al. 2020). The IC₅₀ values are approximately 84.5 µM at 24 h and approximately 46.3 µM with longer exposure in HCT116 cells, and its direct cytotoxicity is comparatively mild despite these complex mechanisms (Jan et al. 2025). Piperine is essential not only for its direct anti-proliferative effects but also because it can be used as an adjuvant to increase the bioavailability and efficacy of other medications.

Colchicine, an alkaloid from *Colchicum pusillum*, has strong anti-proliferative effects through the modulation of the Wnt/β-catenin pathway. In metastatic Colo-741 cells, colchicine treatment increased β-catenin and LGR-5 while suppressing Wnt7a and activating caspase-3-mediated apoptosis. However, the IC50 value of ~ 20 μg/mL at 48 h was accompanied by high toxicity in non-metastatic cells, reflecting poor selectivity (Becer et al. 2019). Compared with piperine, colchicine is more potently cytotoxic but lacks a chemopreventive safety profile. Reflexin A, which is isolated from *Rauvolfia reflexa*, has demonstrated significant anticancer potential with selectivity for malignant cells. In HCT-116 and HT-29 CRC models, Reflexin A triggered apoptosis via caspase-3/7, 8 and 9 activation, induced G_1_ phase arrest, and inhibited cell migration and invasion, with IC_50_ values decreasing from ~ 30 μM at 24 h to ~ 16 μM at 72 h (Fadaeinasab et al. 2020). This profile suggests stronger direct apoptotic and antimetastatic effects than piperine does although the latter results in broader multi-pathway modulation.

Fungal metabolites such as Chaetocochin J from *Chaetomium* represent another class of highly potent alkaloids. With sub-micro-molar IC_50_ values (0.56–0.65 μM), chaetocochin J induces both apoptosis and autophagy by activating AMPK while inhibiting the PI3K/AKT/mTOR pathway, thereby disrupting central metabolic and survival signaling (Hu et al. 2021). While markedly more potent than piperine is, its cytotoxic profile suggests potential therapeutic applications in aggressive CRC contexts, in contrast to piperine’s suitability for long-term chemoprevention owing to its lower toxicity and wider mechanistic range. Caulerpin offers a structurally novel framework with IC50 values of 10–30 μM in HCT116 cells (Mert-Ozupek et al. 2022). Although mechanistic studies remain limited, related compounds indicate that mitochondria-mediated apoptosis involves ROS generation and caspase activation. Compared with piperine, these marine alkaloids are more directly cytotoxic but lack detailed pharmacologic characterization. Piperine, by contrast, benefits from extensive mechanistic evidence across multiple oncogenic pathways.

Unlike these direct cytotoxic agents, berberine, which is derived from *Coptis chinensis*, has a microbiome-centered chemo-preventive mechanism. In an AOM/DSS-induced murine model of colitis-associated CRC, oral berberine (50–100 mg/kg) significantly reduced the tumor burden by remodeling the gut flora. This was associated with elevated short-chain fatty acids, restoration of intestinal barrier proteins (Occludin, ZO-1), reduced lipopolysaccharide levels, and inhibition of the TLR4/NF-κB/IL-6/p-STAT3 axis (Yan et al. 2022). Unlike piperine, which primarily acts within cancer cells by modulating intracellular oncogenic signaling, berberine represents an alternative approach through host–microbiota–immune regulation. In addition to these alkaloids, dentatin, which is isolated from *Clausena excavata*, has shown promising anticancer effects in CRC. In vitro studies using HCT116 and HT29 cells demonstrated that ROS-mediated apoptosis is characterized by the activation of caspase-3/7, 8 and 9, along with the modulation of apoptotic regulators (Bcl-2, Bax). Dentatin also induced G_0_/G_1_ cell cycle arrest and, importantly, triggered the release of Th1-related cytokines, such as IFN-*γ*, IL-2, and TNF-*α*, suggesting dual roles in both apoptosis and immunomodulation. With an IC50 of ~ 19 μM in HCT116 cells, dentatin is more potent than piperine in terms of direct cytotoxicity and offers the additional advantage of enhancing antitumor immunity (Zulpa et al. 2023). In contrast, piperine, although less cytotoxic, influences a wider network of signaling pathways relevant to chemoprevention. To provide a consolidated overview, a summary table (Table 6) has been included highlighting representative alkaloids, their experimental models, signaling pathways, potencies, and comparisons with piperine.

**Table 6:** Comparison of piperine and other alkaloids in CRC treatment

Alkaloid	Type of preclinical study	Animal/cell line model used	Signaling pathways involved	IC50/effective dose	Comparison with piperine	References
Colchicine	In vitro	Colo-320 and Colo-741	Increased β-catenin and LGR-5 expression, activation of caspase-3, and downregulation of Wnt7a, indicating modulation of the Wnt/β-catenin signaling pathway and induction of apoptosis	20 μg/mL	Strong cytotoxic and pro-apoptotic effects but nonselective toxicity; Piperine is less cytotoxic but has broader chemopreventive activity	(Becer et al. 2019)
Reflexin A	In vitro	HCT-116, HT-29, normal CCD-841 cells	Activated caspase-3/7, caspase-8, and caspase-9, caused G1 cell cycle arrest, and inhibited cell migration and invasion, thereby suppressing tumor growth and metastatic potential	30.24 μM (24 h); 16.06 μM (72 h)	More potent apoptosis inducer than piperine with selectivity for cancer cells; Piperine has broader modulation but lower direct cytotoxicity	(Fadaeinasab et al. 2020)
Chaetocochin J	In vitro	RKO, HCT116, SW480	Induced apoptosis through caspase-3 activation and PARP cleavage while simultaneously inducing autophagy, evidenced by increased LC3-II and decreased p62 levels, mediated by AMPK activation and inhibition of the PI3K/AKT/mTOR pathway	0.56–0.65 μM	Far more potent than piperine (subμM vs. tens of μM); induces both apoptosis and autophagy. Piperine works via multi-target modulation	(Hu et al. 2021)
Caulerpin	In vitro	HCT116	Induced mitochondria-mediated apoptosis through elevated ROS generation and caspase activation	10–30 μM	More potent than piperine but less mechanistically studied. Piperine better-documented for multipathway targeting	(Mert-Ozupek et al. 2022)
Berberine	In vivo	C57BL/6 J mice	Modulating gut flora, leading to increased production of short-chain fatty acids (butyrate, acetate, propionate), decreased lipopolysaccharide levels, restoration of barrier proteins (Occludin and ZO-1), and inhibition of the TLR4/NF-κB/IL-6/p-STAT3 signaling axis	50–100 mg/kg orally	Unlike piperine (direct signaling modulation in cancer cells), berberine works via microbiota remodeling + SCFA metabolism, reducing tumorigenesis	(Yan et al. 2022)
Dentatin	In vitro	HCT116 and HT29	Induced ROS-mediated apoptosis with activation of caspase-3/7, -8, and -9, caused G_0_/G_1_ cell cycle arrest, and enhanced immune response by increasing Th1 cytokines (IFN-γ, IL-2, TNF-α), along with downregulation of Bcl-2 and upregulation of Bax	19.4 μM	More potent apoptosis inducer than piperine, with added immunomodulatory activity (Th1 cytokine induction). Piperine exerts multi-target effects but weaker cytotoxicity	(Zulpa et al. 2023)

These findings highlight the spectrum of alkaloid activity in CRC. Colchicine, Reflexin A, Chaetocochin J, and Caulerpin act as direct cytotoxic agents with varying potencies and selectivities, whereas berberine exerts its anticancer effects by altering the tumor microenvironment via microbiota remodeling. Piperine falls in between—its direct cytotoxicity is modest compared with that of potent alkaloids such as chaetocochin J, but its strength lies in multi-pathway modulation and chemo-preventive potential with a favorable safety profile. These findings position piperine as a complementary nutraceutical candidate that may be especially valuable in long-term CRC prevention or in synergistic regimens.

## Strategies to improve the therapeutic efficacy of piperine

Despite the wide range of pharmacologic characteristics of piperine, its poor water solubility, low bioavailability, and quick metabolic breakdown limit its application in clinical settings (Mitra et al. 2022). Recent developments in nanotechnology have made it easier for researchers to develop delivery methods based on piperine, which are intended to circumvent these restrictions. Piperine’s solubility, cellular uptake, and controlled release have all been improved by the development of a variety of nanoparticles, including polymeric, liposomal, micellar, metallic, and solid lipid-based forms. By facilitating targeted drug distribution and enhancing the ability of piperine to cross biologic membranes, these nano-carriers reduce systemic toxicity (Bose et al. 2023; de Oliveira et al. 2022; Imam et al. 2021; Kiranmayee et al. 2023; Raghunath et al. 2024).

Compared with pure piperine, piperine nanoemulsions have been demonstrated to increase intestinal permeation and improve drug release over those of pure piperine (Alshehri et al. 2023). Compared with conventional suspensions, piperine-loaded lipid‒polymer hybrid nanoparticles (PPN‒LPHNPs) exhibit excellent colloidal stability, sustained drug release for up to 24 h, and a 6.02-fold increase in intestinal permeation. These attributes result in more than a fourfold increase in oral bioavailability and, importantly, superior cytotoxic effects against the breast cancer cell lines MDA-MB-231 and MCF-7 (Kazmi et al. 2022). Similarly, hydroxyapatite nanoparticle-based systems provide targeted delivery to colon cancer cells, with slow release kinetics, functionalization for folic acid targeting, and notably enhanced tumor cell inhibition while minimizing toxicity to normal cells (AbouAitah et al. 2020). Combination nano-formulations that incorporate curcumin and piperine have demonstrated the selective suppression of prostate cancer cell migration and proliferation, reducing off-target toxicity in healthy cells. These nanocarrier systems enhance cellular uptake, stability, and tumor targeting and allow for lower doses with sustained anticancer activity (Yakubu et al. 2025).

Piperine analogs, which are structural modifications of piperine, have also been studied to enhance its pharmacologic profile and bioavailability and reduce toxicity (Joshi et al. 2023). Various piperine analogs have been designed to improve water solubility, metabolic stability, and biologic activity by altering their functional groups or isomeric forms. Some analogs aim to retain the bio-enhancing properties of piperine while minimizing off-target effects, thereby providing better therapeutic indices. These analogs, combined with advanced delivery systems, represent a promising approach to increase the clinical potential of piperine-derived compounds (Joshi et al. 2023; Tripathi et al. 2022). Research on piperine analogs has focused on improving their anticancer properties and managing drug resistance. Low-molecular-weight analogs, such as Pip1 and Pip2, have been engineered to potently inhibit P-glycoprotein (P-gp), an efflux transporter implicated in multidrug resistance in cancers. These analogs significantly enhanced the accumulation and efficacy of chemotherapeutic agents (vincristine, colchicine, and paclitaxel) in P-gp-overexpressing cervical and colon cancer cells, resulting in up to a ninefold reversal of drug resistance, comparable to that of standard P-gp inhibitors such as verapamil. Molecular docking and simulation studies further confirmed that these analogs outcompeted piperine in terms of binding affinity for P-gp. In prostate cancer investigations, the piperine analog Pip2 displayed the strongest inhibition of the Akt1 pathway, with a high predicted binding affinity and potentially enhanced chemotherapeutic impact compared with native piperine. These analogs either retain or enhance cytotoxic activity while potentially reducing off-target effects or toxicity (Prakash 2023; Syed et al. 2017).

## Conclusion and future perspectives

Alkaloids are a structurally diverse class of natural compounds with notable anticancer potential. Piperine has emerged as a promising candidate for colorectal cancer (CRC) therapy owing to its anti-inflammatory, antioxidant, and multi-target mechanisms. Preclinical evidence highlights its ability to induce apoptosis; regulate autophagy; suppress oncogenic signaling pathways, such as Wnt/β-catenin, STAT3/Snail-EMT, and PI3K/Akt/mTOR pathways; and modulate inflammatory and oxidative stress responses, collectively resulting in reduced cell proliferation, angiogenesis, invasion, and metastasis. In addition, piperine functions as a natural bio-enhancer, enhancing the bioavailability and therapeutic efficacy of chemotherapeutics, radiotherapy, and nutraceuticals, positioning it as a valuable adjuvant in cancer therapy. Despite these promising findings, several gaps remain that hinder translational progress. However, its poor aqueous solubility, rapid metabolism, low oral bioavailability, and short half-life limit its systemic stability. Safety concerns regarding higher doses, particularly reproductive toxicity and gastrointestinal irritation, necessitate careful dose optimization. Moreover, most studies remain confined to in vitro and a few rodent models, with a lack of standardized dosing protocols, validated CRC models, and detailed pharmacokinetic and pharmacodynamic profiles. The absence of well-structured clinical trials is one of the most significant barriers to clinical translation, with potential challenges of resistance and reduced efficacy with long-term piperine treatment.

Therefore, future research should focus on overcoming these challenges via a multipronged approach. Standardized preclinical investigations using validated CRC models are essential for establishing reproducibility, defining dose‒response relationships, and confirming safety. Comprehensive pharmacokinetic and pharmacodynamic studies are needed to better understand absorption, metabolism, drug–drug interactions, and drug–nutrient interactions. Pharmaceutical innovations, such as nanotechnology-based formulations, including liposomes, polymeric nanoparticles, micelles, and lipid-based carriers, offer practical solutions for improving solubility, stability, and bioavailability. In parallel, structural analogs and derivatives of piperine should be explored to increase its potency, specificity, and resistance-modulating capacity. Given its role as a bio-enhancer, systematic evaluations of piperine in combination with chemotherapeutics, radiotherapy, immunotherapy, and nutraceuticals, such as curcumin, resveratrol, and cannabinoids, are warranted to harness its synergistic potential. Importantly, combination studies with other alkaloids should also be prioritized as multi-alkaloid formulations may offer additive or synergistic modulation of oncogenic pathways while reducing resistance. Early-phase clinical trials (Phase I/II) are needed to establish the safety, tolerability, and preliminary efficacy of this combination therapy in CRC patients, which will pave the way for larger multicenter studies. By integrating molecular insights, advanced delivery systems, and rigorous clinical validation, piperine can move beyond its current preclinical promise and be developed as a safe and effective adjunct for colorectal cancer management.

## Data Availability

This review article analyses data from previously published studies. All data supporting the findings of this review are available within the cited references. All the figures have been acquired from the respective journals.
